# Opportunities, challenges, and ethical implications of online pediatric nutrition consultation: a nursing perspective

**DOI:** 10.3389/fped.2026.1784261

**Published:** 2026-03-16

**Authors:** Ling-ling Ma, Ke-xin Wang

**Affiliations:** 1 Department of Outpatient, The Second Affiliated Hospital of Mudanjiang Medical University, Mudanjiang, China; 2 Department of Pediatrics, Hongqi Hospital Affiliated to Mudanjiang Medical University, Mudanjiang, China

**Keywords:** care coordination, digital health, health equity, nursing ethics, pediatric nutrition, telehealth

## Abstract

Against the backdrop of rapid global expansion in digital healthcare and growing demand for child nutrition services, this perspective article examines the emerging model of online pediatric nutrition consultation. We argue that while this model offers significant potential to improve accessibility and efficiency, it faces multidimensional challenges. These include technical barriers such as the digital divide and limitations in remote assessment; professional practice issues like constrained communication and inadequate adaptation of clinical tools; and ethical complexities related to privacy, accountability, and equity. To support its responsible development, this perspective article applies a nursing ethics framework—centered on autonomy, non-maleficence, beneficence, and justice—to analyze these challenges. We further propose integrated strategies focusing on ethics-guided practice, technological standardization, professional competency development, and supportive policy ecosystems to ensure that innovation aligns with safe, equitable, and person-centered care.

## 1 Introduction

The global trend towards digital healthcare is fundamentally transforming service delivery (1). Concurrently, issues of pediatric nutrition and health—such as malnutrition, obesity, and feeding disorders—present growing public health concerns. Within this context, online pediatric nutrition consultation has emerged as a significant development (2, 3). This growth is propelled by supportive regulatory frameworks for digital health, advancements in telecommunication technologies, and increasing parental demand for accessible, expert guidance (4). The use of telehealth in pediatric care—encompassing nutrition guidance, chronic disease management, and obesity interventions—has expanded considerably (5). Nevertheless, its implementation faces notable practical constraints, such as limitations in remote physical assessment, dependence on caregiver-reported data, potential communication barriers, and inequitable access stemming from technological and socioeconomic disparities (6). Examining this model is therefore critical for enhancing service quality, optimizing resources, and improving child health outcomes (7).

The application of online consultation to pediatric nutrition involves distinct challenges. Pediatric assessment relies heavily on nuanced data: growth metrics, detailed dietary histories, and observations of feeding behavior, which are more difficult to obtain and interpret remotely (6, 8). Furthermore, the process necessitates parents as active intermediaries, altering the traditional practitioner-patient dynamic (8). This expanded role for healthcare providers—encompassing clinician, educator, and coordinator—carries heightened ethical responsibilities (9, 10). These include ensuring accurate assessment in a virtual context, safeguarding minor patient privacy, maintaining professional boundaries, and exercising prudent clinical judgment despite potential informational gaps (11).

**Figure 1:**
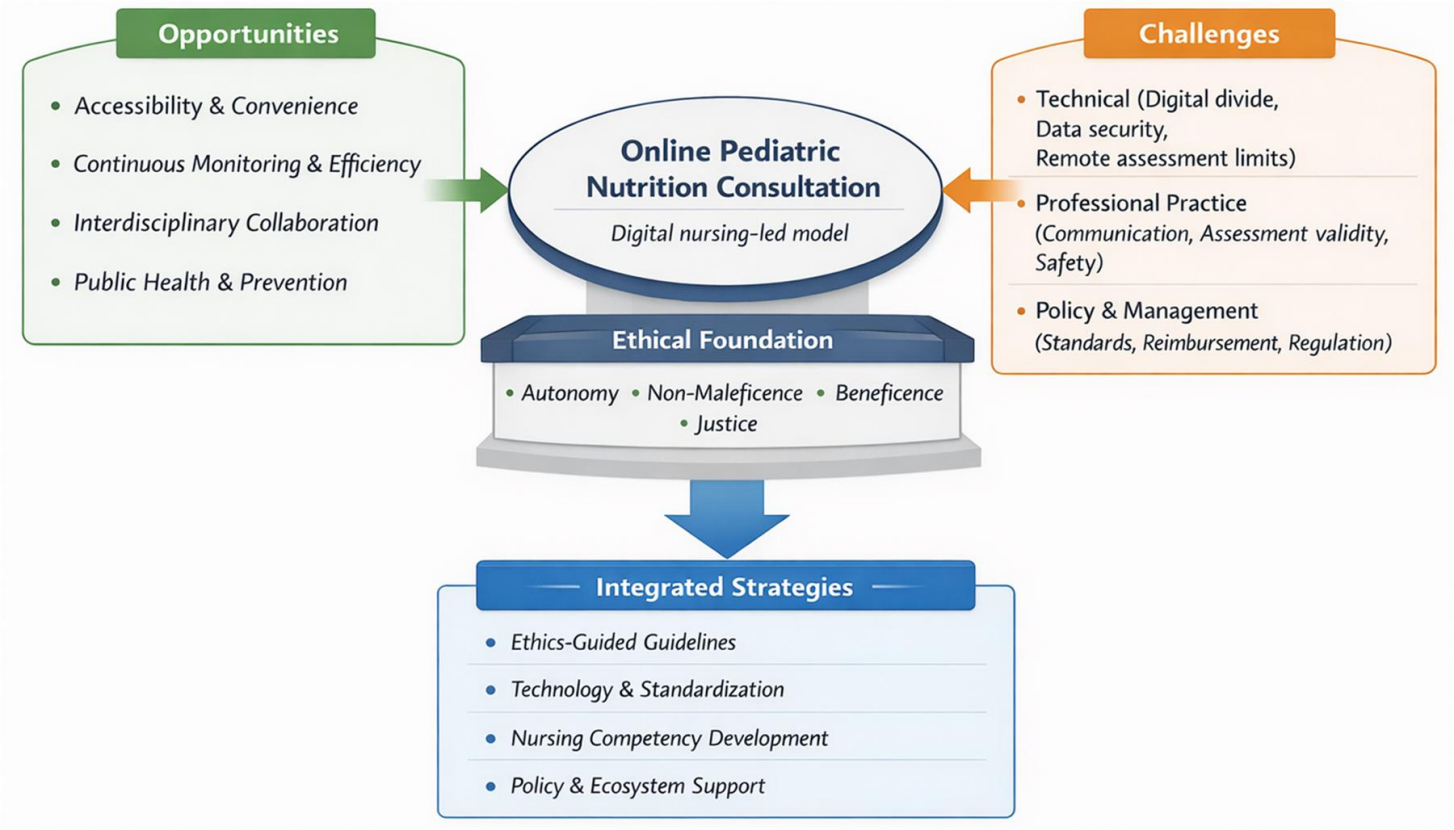
Conceptual framework of opportunities, challenges, and ethical principles in online pediatric nutrition consultation.

This study adopts the format of a perspective article, distinguishing it from systematic or scoping reviews. Its central purpose is to present a conceptual discussion of online pediatric nutrition consultation from a nursing ethics standpoint, rather than a comprehensive synthesis of existing evidence. The cited literature was selected for its representativeness and authority to support ethical reflection and practice-oriented discussion. Consequently, this work does not employ formal evidence appraisal or comparative methodological assessment. The article proceeds by critically engaging with this mode of digital healthcare. It opens by identifying the principal advantages and prospective value of remote pediatric nutrition services. Subsequently, it addresses the salient clinical, technical, and communication-related difficulties inherent in such services. A substantial portion is devoted to examining the accompanying ethical considerations, resulting in the formulation of a proposed structure for ethically sound nursing practice. Finally, the article presents a series of prospective recommendations designed to guide policy, clinical protocols, and subsequent inquiry in this emerging area, as summarized in Figure 1.

## 2 Opportunities in online pediatric nutrition consultation

### 2.1 Enhanced accessibility and convenience

Online pediatric nutrition consultation significantly improves service accessibility by overcoming geographical limitations (12). It provides vital support to families in remote or underserved areas, as well as to those with mobility constraints (13). The model also offers considerable scheduling flexibility, allowing consultations to be arranged outside conventional hours (6). This reduces travel time, waiting periods, and overall barriers to accessing professional guidance (14).

### 2.2 Improved efficiency in health management

Digital platforms facilitate more precise and continuous health monitoring (15). Parents can easily record and share their child's dietary intake, symptoms, and growth measurements through dedicated applications, enabling real-time tracking of developmental progress (16). This continuous data flow supports better clinical assessment and early intervention (17). Furthermore, the integration of multimedia educational materials—such as instructional videos and visual guides—enhances parental comprehension and adherence to nutritional recommendations more effectively than traditional methods (18).

### 2.3 Facilitated interdisciplinary collaboration and family engagement

Online systems promote coordinated care by allowing seamless communication among nutritionists, pediatricians, and other healthcare providers (19). This ensures a consistent and integrated approach to patient management (20). Importantly, the model actively involves parents and primary caregivers in the care process (21). By increasing their knowledge and self-efficacy, it strengthens their ability to implement dietary interventions, thereby improving long-term adherence and health outcomes (12).

### 2.4 Public health and preventive potential

From a public health perspective, online consultation platforms enable scalable nutrition screening and early identification of at-risk populations (22). Widespread implementation can support large-scale preventive initiatives (6). Additionally, securely aggregated and anonymized population data can inform nutritional epidemiology research, guide the evaluation of intervention programs, and contribute to evidence-based public health policy and planning (23).

## 3 Key challenges in online pediatric nutrition consultation

### 3.1 Technical challenges

Online pediatric nutrition consultation faces significant technical barriers. The “digital divide”—inequalities in internet access, device availability, and digital literacy—limits access for families in underserved or remote regions, creating disparities in service availability (24). Data security and patient privacy remain critical concerns, as the transmission and storage of sensitive health information require stringent safeguards (25). Moreover, the remote format inherently restricts physical assessment, making accurate growth measurements and direct clinical observation challenging, which may compromise the depth of nutritional evaluation (26, 27).

### 3.2 Professional practice challenges

The virtual environment imposes constraints on clinical interaction. Reduced non-verbal communication can hinder the clinician's ability to assess caregiver comprehension, emotional state, or environmental factors, potentially affecting therapeutic rapport and trust (28). Furthermore, traditional dietary assessment methods, such as 24 h recalls, may be less reliable when conducted remotely without visual aids or in-person guidance (29). A notable clinical gap is the lack of established protocols for identifying and managing urgent nutrition-related issues—such as severe feeding refusal or suspected allergic reactions—in a remote setting, raising potential safety concerns (30).

### 3.3 Management and policy challenges

The absence of standardized quality frameworks and evidence-based practice guidelines for online pediatric nutrition services results in inconsistent care quality and complicates regulatory oversight (6, 31). Reimbursement structures also lag behind service development; most public and private insurers do not consistently cover these consultations, undermining their financial sustainability and widespread adoption (32, 33). Furthermore, clear professional standards defining the scope of practice, documentation requirements, and ethical responsibilities for providers in digital health contexts are insufficiently developed, creating legal and operational uncertainties (31). Addressing these systemic issues requires coordinated policy development, professional guideline establishment, and structured support from healthcare institutions.

## 4 An ethical analysis framework for nursing practice

From a nursing perspective, online pediatric nutrition consultation functions as a hybrid practice model that combines clinical assessment, family education, care coordination, and ethical decision-making (6, 34, 35). In this model, nurses act as key intermediaries between families and digital health systems, undertaking roles that go beyond the scope of traditional in-person care.

### 4.1 Application of core ethical principles in online pediatric nutrition consultation

The transition to online service delivery necessitates a careful re-examination of core nursing ethics. Respect for autonomy involves navigating the balance between parental decision-making and the evolving capacity of the child (36, 37). The virtual setting complicates obtaining meaningful assent from the child and ensuring parents are fully informed (36, 38). Non-maleficence requires vigilance against potential harms inherent to remote care, such as diagnostic inaccuracies from limited data, inappropriate nutritional advice, or anxiety induced by digital information overload (36). Beneficence obligates providers to actively leverage digital tools—like tailored educational content and continuous monitoring—to optimize nutritional outcomes. Finally, justice calls for deliberate service design to mitigate the “digital divide,” ensuring equitable access across socioeconomic, geographic, and technological barriers (38, 39).

### 4.2 Specific ethical issues in digital nursing practice

Beyond the foundational principles, online pediatric nutrition care surfaces distinct ethical complexities (40). Relational ethics is challenged by the constraints of virtual interaction, which can inhibit the development of trust and therapeutic rapport, and limit the nurse's ability to provide nuanced emotional support (41). Confidentiality demands robust data security protocols to protect sensitive pediatric health information, while also navigating complex family dynamics regarding information sharing among caregivers (35). Professional responsibility requires clear delineation of the nurse's role in a hybrid care model. This includes defining the scope of online practice, establishing formal pathways for referral to in-person services, and clarifying accountability, thereby safeguarding both patient safety and professional integrity in a digitally mediated environment (36, 40).

## 5 Integrated strategies addressing opportunities, challenges, and ethics

To address the multifaceted challenges inherent in online pediatric nutrition consultation and to fully realize its benefits, a coordinated strategy integrating technology, practice, ethics, and policy is required (Table 1).

**Table 1:** Alignment of opportunities, challenges, ethical considerations, and recommended strategies in online pediatric nutrition consultation.

Dimension	Key issues/features	Ethical implications	Corresponding strategies
Accessibility	Remote access, flexible scheduling	Justice, equity	Digital inclusion policies
Health management	Continuous monitoring, mHealth tools	Beneficence, non-maleficence	Validated tools, safety protocols
Professional practice	Communication limits, role expansion	Professional responsibility	Training, scope clarification
Data & privacy	Data transmission, storage	Confidentiality, autonomy	Secure platforms, consent standards
Policy & system	Lack of standards, reimbursement	Justice, accountability	Guidelines, insurance coverage

### 5.1 Develop ethics-guided practice guidelines

A specialized Code of Ethical Practice for Online Pediatric Nutrition Consultation should be established, grounded in core ethical principles (25). This code should standardize procedures for obtaining informed consent—with particular attention to developmentally appropriate methods for securing child assent—and define clear protocols for data privacy, security, and the permissible use of electronic health records (42, 43). Additionally, evidence-based, age-specific communication frameworks should be created to guide practitioners in respectfully engaging children according to their developmental stage (44).

### 5.2 Strengthen technology and standardization

Technology development should be purposeful and secure. This includes the validation and implementation of pediatric-specific remote assessment tools (e.g., reliable digital dietary instruments) and the establishment of secure, compliant data platforms (45, 46). Concurrently, standardized clinical safety protocols should be developed, detailing procedures for identifying urgent situations during remote consultations and ensuring seamless referral pathways to in-person care (47, 48).

### 5.3 Enhance nursing professional competency

The quality of online care depends directly on provider proficiency (49). Nursing education and continuing professional development should incorporate training in digital health literacy, remote assessment, and effective virtual communication—including techniques for building rapport and delivering empathetic support in a digital medium (50, 51). This training should be grounded in a clear ethical framework that provides practical guidance for nurses in structuring online consultations, determining appropriate criteria for escalating to in-person care, and maintaining therapeutic relationships within virtual settings. Concurrently, training curricula should emphasize interdisciplinary collaboration and ethical reasoning in complex, technology-mediated scenarios (35). The findings from such integrated training and ethical practice may subsequently inform the refinement of nursing education standards, the development of institutional protocols, and the evolution of professional guidelines, particularly for specialized fields such as pediatric digital nutrition services.

### 5.4 Optimize policy and ecosystem support

Sustainable integration requires supportive policy and infrastructure. Policymakers, in collaboration with professional bodies, should define clear service standards, scope of practice, and accountability mechanisms for online pediatric nutrition care (6, 52). Reimbursement models, including public and private insurance coverage for validated services, should be established to ensure financial viability (53, 54). At a systemic level, public investment in digital infrastructure and literacy is essential to bridge the digital divide and ensure equitable access for all families (55).

## 6 Summary

Online pediatric nutrition consultation represents a significant advancement in digital healthcare, offering substantial potential to improve the accessibility, continuity, and efficiency of child nutrition services. However, this model presents interconnected challenges across technical, clinical, regulatory, and ethical domains. Ethical considerations, in particular, require explicit frameworks and guidelines to ensure safe and equitable practice. Nurses play an indispensable role in this evolving landscape, necessitating both technical proficiency in digital tools and a steadfast commitment to maintaining therapeutic relationships, safeguarding patient welfare, and upholding ethical standards in a virtual environment. The effectiveness of online consultation fundamentally depends on this balanced integration of competence and care.

To ensure the responsible growth of this field, further rigorous research is needed to evaluate the efficacy, safety, and long-term outcomes of various online service models. This evidence is crucial for establishing best practices and standards. Moreover, as technology and service delivery continue to evolve, the core principle of patient- and family-centered care should remain paramount. The future of pediatric nutrition consultation lies in aligning innovation with this foundational ethic, ensuring that digital tools enhance rather than undermine compassionate, individualized care. Achieving this balance is essential for harnessing the full potential of digital health to support the well-being of children and families.

## Data Availability

The original contributions presented in the study are included in the article/Supplementary Material, further inquiries can be directed to the corresponding author.
